# *“It’s Worth Its Weight in Gold”*: Patient Reported Experience Measures of a National NHS-Funded Community Pharmacy-Based Common Ailments Service

**DOI:** 10.3390/pharmacy14050112

**Published:** 2026-08-03

**Authors:** Efi Mantzourani, David McRae, Egerton Hunter, Grace Naylor, Emma Hinks, Andrew Evans, Rebecca Cannings-John

**Affiliations:** 1 Cardiff School of Pharmacy and Pharmaceutical Sciences, Cardiff University, Cardiff CF10 3NB, UK; huntere6@cardiff.ac.uk (E.H.); naylorg1@cardiff.ac.uk (G.N.); 2 Digital Health and Care Wales, National Health Service Wales, Cardiff CF11 9AD, UK; 3 Primary Care Services, Welsh Government, Cardiff CF10 3NQ, UK; david.mcrae@gov.wales (D.M.); emma.hinks@gov.wales (E.H.); andrew.evans@gov.wales (A.E.); 4 Centre for Trials Research, Cardiff University, Cardiff CF14 4YS, UK; canningsrl@cardiff.ac.uk

**Keywords:** common ailments service, patient-reported experience measures, community pharmacy, minor ailments, PREMs, healthcare quality

## Abstract

The NHS-funded Common Ailments Service (CAS) in Wales enables community pharmacists to manage 28 common ailments by providing advice and/or medication free of charge without the need for patients to book an appointment. The aim of this study was to evaluate the patient experience of the CAS using patient reported experience measures (PREMs) and determine the feasibility of digital data capture collected directly from patients through a pharmacist-led manual recruitment model. The study used prospective, primary data collected through an online survey accessible via a Quick Response code and census recruitment. A total of 3660 surveys were included in the analysis, completed between 27 October 2025 and 31 March 2026. Overall experience was rated as excellent by 95.1% of participants. A theory-based framework analysis of 1499 free-text comments across the six domains of healthcare quality confirmed the high standard of care provision, primarily influenced by consultation quality, convenience, accessibility, and short waiting times. It also identified targeted areas for improvement, such as the need for seamless referrals from other areas of primary care and consistent availability of the service across all pharmacies. While digital PREMs captured high-quality insights, the pharmacist-led recruitment model reached a “feasibility ceiling”, with an overall response rate of 3.2%.

## 1. Introduction

Internationally, community pharmacists have seen a notable shift in professional responsibility, moving from being limited to traditional dispensing roles to becoming a first point of contact for many minor ailments and uncomplicated presentations of infections, such as sore throat and urinary tract infections [1]. Prudent healthcare is a strategic approach within the Welsh NHS designed to ensure sustainability and quality by delivering only necessary, evidence-based care, reducing inappropriate variation in clinical practices, and ensuring patients are being seen by the most appropriate healthcare professional [2]. In line with this strategic direction, the Common Ailments Service (CAS) was introduced in 2013, enabling community pharmacists to manage 28 common ailments by providing advice and/or medication free of charge funded by NHS Wales. Since its initial roll-out the service has expanded substantially, with more than 462,000 consultations occurring in 2024–2025, an increase of 34.4% from 2022–2023 and more than six times (506.6%) as many consultations as five years ago [3].

The initial evaluation of the service in 2015, the results of which led to its national roll-out and commissioning, used data from CAS registration and consultation data, GP prescription data, and information from the Secure Anonymised Information Linkage databank [4]. This quantitative data were supplemented by surveys, focus groups and interviews with pharmacists, general practitioners, and stakeholders. While attempts were made to capture patient engagement with and experience of the service, only sixteen patient responses were received; fourteen of the sixteen patients responding not having used the service. Due to concerns around the limited sample size and its representativeness, the evidence captured directly from patients was not included in the evaluation. Since then, the service has expanded to include conditions requiring enhanced clinical assessment, with sore throat test and treat (STTT) and urinary tract infection (UTI) management initially commissioned nationally as non-mandatory elements in 2022 and 2024, respectively, before being consolidated into the mandatory CAS service offer in 2025. Follow-up evaluations of the service have explored service uptake statistics, clinical outcomes and patient experience for STTT and UTI only [5,6], and the relationship between the provision of CAS as a whole and socioeconomic deprivation [7,8].

The use of patient-reported experience measures (PREMs) is regarded as critical to evaluating the quality and performance of health services and is largely standard of practice in many healthcare systems, including the United Kingdom. PREMs are instruments that capture a patient’s experience of receiving care, commonly used across NHS services to provide insight or action to promote improvement and patient-centred care aligning with the principles set out in the NHS Wales People’s Experience Framework [9]. Systematically collecting these measures allows healthcare organisations to improve service delivery and promote more informed decision-making [10]. As part of the current CAS, a limited set of data related to what patients would have done had CAS not been available and whether patients tried to see a GP prior to presenting in the pharmacy is captured as part of the service. This information is entered on Choose Pharmacy, the community pharmacy national IT platform where all pharmacy services are recorded in Wales. The questions are limited; do not capture patients’ views on their experience of the consultation, how the experience aligns with their expectations of the service, or impacts their perceived future health-seeking behaviour; and are asked and entered into the Choose Pharmacy platform by the pharmacist who carried out the consultation, introducing the potential for social desirability and reporting bias.

With the increasing focus on the shift of safe management of uncomplicated conditions from general practice to community pharmacies, and with the potential for more ailments to be added to CAS in the future, there is a need to evaluate patient experiences across all aspects of CAS consultations at a large scale, with information reported directly by patients used to elucidate whether the service is meeting its policy objectives and meeting the needs of the citizens of Wales who access the service.

The use of an online data collection system where patients can enter data directly is already used elsewhere in Wales. The National Survey for Wales has been developed and tested for use across services, with patients able to complete questionnaires through online data collection to capture feedback on their care. Using a similar approach for the CAS could enable immediate, patient-led feedback, supporting both data integrity and scalability across the extended network of around 700 community pharmacies across Wales. Digital patient-reported experience measure collection in primary care is technically feasible, with the literature reporting patient completion rates ranging from 6.4% to 100% depending on implementation approach, though operational integration into routine care workflows remains challenging [11,12,13,14,15]. An important aspect of implementation is to establish a proof of concept and test its feasibility to further inform a model that can be scaled up and enable continuous sustainable evaluation, replacing current information collection by pharmacists.

The aim of the present study was, therefore, to evaluate the patient reported experience measures (PREMs) of the CAS and determine the feasibility of using PREM data collected directly from patients through an online survey accessed by QR codes supplied by pharmacists following consultations and through pharmacy-based promotional materials at the point of care.

## 2. Materials and Methods

### 2.1. Study Design

A positivism research paradigm was followed, with a quantitative methodology to evaluate the experience of patients with the Common Ailments Service in Wales and to describe the feasibility of capturing experience measures via a pharmacist-led recruitment model from as many patients as possible. The study was based on prospective, primary data collection with a survey as a data collection tool.

The objectives of the study were as follows:

- Determine the feasibility of using a pharmacist-led manual recruitment with online data collection for continuous sustainable evaluation of PREMs, assessed by response rate.
- Describe the patient characteristics and factors related to their engagement with the service.
- Describe the patient experience of the service in relation to shared decision-making.
- Describe the patient experience of the service in relation to advice pharmacists provided on managing current symptoms, safety netting, and preventing future symptoms.
- Describe how the patient experience during a service consultation impacts patient-perceived future health-seeking behaviour.

### 2.2. Project Population, Sampling, and Recruitment

Data were collected between 27 October 2025 and 31 March 2026, as part of the wider Winter Incentive Scheme (WIS), a non-compulsory annual scheme agreed between the Welsh Government, Community Pharmacy Wales, and the Health Boards in Wales [16]. For pharmacists participating in the WIS, no sampling of patients was carried out; instead, a census approach was adopted and all patients who had a CAS consultation were invited to take part in the survey. If a patient lacked capacity but was accompanied by a carer, the carer was asked to complete the survey on the patient’s behalf. Assessment of patients’ capacity was conducted as part of routine service provision.

A pack containing a credit-card-sized handout and A5 laminated sheets, both containing the QR code to the patient information sheet (PIS) and the electronic survey, were distributed to pharmacies taking part in the CAS service. A cover letter explained the purpose and background of the study and provided information on how the provided resources should be used. At the end of each CAS consultation, pharmacists participating in the WIS were asked to provide every patient (or their carer) with brief information about the study during their consultation and encourage them to provide feedback on their experience by completing the survey. Pharmacists were asked to provide patients (or their carers) with the QR code to access the electronic survey, either by referring to the code on the A5 poster or by providing a credit card sized handout containing the code; however, there was no mechanism to distinguish whether a response was provided by a patient or their carer in the survey. This could be delegated to pharmacy team members so long as they were able to provide the patient with the relevant information. No option for completion of a paper survey was provided, due to the high cost associated. All study and promotional materials were available in English and in Welsh. No incentives for participants were used for this study.

As part of the WIS, community pharmacists were asked to endorse the consultations whereby a patient had been invited to take part in the survey with the code “PREMs” on *Choose Pharmacy*. The overall number of consultations endorsed with the code PREMs for the duration of the study (assumed to equate to the population invited to complete the survey) were obtained from Digital Health and Care Wales and used to calculate the response rate. The total number and consultation characteristics during the same time period were also obtained and used to compare the final survey sample with the total population cohort and explore representativeness.

### 2.3. Sample Size

We assumed just under 147,000 CAS consultations would take place between 1 November 2025 and 31 March 2026 (based on 2023/2024 data from the Welsh Government annual report on community pharmacy services [16]), and that there would be a 2.5% response rate to the survey (*n* = 3675). Given the predicted sample size, it was expected there would be enough survey responses for most ailments to make robust estimates of patient experience of the CAS consultation, e.g., we estimated there would be 290 to 550 consultations (8–15% of all consultations) for warts and verrucae, vaginal thrush, threadworms, teething, and sore throat and 100 to <290 consultations (3–8%) for indigestion and reflux, oral thrush, and scabies. For analyses using the chi-squared test of independence, we expected that all expected cell counts would meet the recommended thresholds of 5 or more.

### 2.4. Data Collection Tool

A version of the survey in English was designed in collaboration with the Welsh Government and members of the public. Its development was based on a combination of the literature, including the national PREMs [9] and stakeholder-informed phrasing of questions around the objectives of the study. Data were captured using a combination of Likert-scale and multiple-choice questions. Additional clarification was sought from patients when the option “other” was selected in specific questions. A free-text question at the end invited feedback and any additional comments patients wanted to add. Once developed, the survey was piloted with further members of the public (*n* = 13), and suggestions in relation to language and the order of questions were actioned for the final version. The final version of the survey in English was then translated in Welsh.

### 2.5. Data Collection, Processing, and Analysis

Responses submitted by patients digitally using the QR code were entered in Online Surveys^®^ (Version 3),which was used to host the electronic survey. Data from Online Surveys^®^ was imported into Excel^®^ (Version 2606) for quality assurance. Responses in Welsh were translated and merged with the English data before processing and analysis. A 10% validation check was completed to assure the quality of the data transfer.

Frequencies and percentages were used to describe patient demographics, service use, and satisfaction. Chi-squared tests were used to explore associations between overall satisfaction with the CAS consultation, expectations of the CAS service, age groups, and ailments, with the results of the chi-squared test presented alongside the *p*-value. All analyses were conducted using IBM SPSS Statistics v27.0.

Following familiarisation with the free-text responses, data were coded inductively and an initial content analysis was completed. Within the principles of implementation science, inductively coded data were mapped against the framework of the six domains of healthcare quality by the Institute of Medicine [17] to explore the impact the service has had on patients’ quality of care. This framework was used as a structured way of assessing quality across recognised dimensions (safe, effective, patient-centred, timely, efficient, and equitable), which closely align with the Welsh NHS Duty of Quality’s focus on improving outcomes, patient experience, and safety [18]. Mapping the data in this way therefore enabled the consideration of how the service contributes to statutory quality expectations in NHS Wales, as well as identifying potential gaps between current provision and best practice. To ensure that relevant concepts not encompassed by the framework were not overlooked, a further inductive analysis was undertaken using the approach outlined above. To increase validity of the analysis, coding was completed independently by three researchers (E.M.; G.C.; E.H.), and any disagreements were discussed and resolved within the group.

### 2.6. Ethical Considerations

A detailed PIS was available at the start of the survey. No identifiable patient details were collected as part of this study. The survey responses were completely anonymous and there was no way of linking any of the information back to participants. Patients were asked to confirm that they have read the PIS, and that they understand that completion of the questionnaire implied consent. The study received ethical approval by the Cardiff School of Pharmacy and Pharmaceutical Sciences Research Ethics Committee (reference: 2526-01; date of approval: 18 June 2025).

## 3. Results

### 3.1. Overall

A total of 671 out of 685 pharmacies participated in the WIS (97.9%). Between 27 October 2025 and 31 March 2026, a total of 240,058 CAS consultations were recorded on the Choose Pharmacy platform, of which 114,216 (47.6%) were endorsed with “PREMs”. A total of 3842 surveys were completed during this time (English: *n* = 3763; Welsh: *n* = 79). Of those, 182 were excluded from the study as participants either clicked on the Welsh QR code by mistake and could not understand the survey (*n* = 11) or the ailment the consultation was for, as added in the option for “other”, was not one available specifically in CAS (e.g., influenza vaccination, ear conditions, contraception) (detailed breakdown in Supplementary Table S1). The response rate was 3.2% (3660/114,216), with a peak in the first week of the survey’s introduction (16.7%) declining to 1.4% in March (Table 1).

The majority of eligible responses were provided from patients between 25–64 years of age (*n* = 2280, 62.3%), and the conditions which received the most completed responses were sore throat (*n* = 594, 16.2%), urinary tract infection (*n* = 561, 15.3%), dermatitis (dry skin) (*n* = 426, 11.6%), and conjunctivitis (*n* = 401, 11.0%) (Table 2). Patients completing the survey (responders) were broadly representative of the total consultations for the same period in terms of age and conditions, with some exceptions (Table 2). Patients completing the survey were more likely to be older apart from those aged 75+ years old, with fewer responses from those aged 0–17 years-old (16.2% vs. 26.9%). Responders were more likely to complete the survey for certain conditions such as urinary tract infections (15.3% survey responders vs. 6.7% CAS consultations) and sore throats (16.2% vs. 13.6%) and less likely to consult for threadworm (3.6% vs. 10.6%) and scabies (3.0% vs. 7.8%). In terms of service outcomes, survey responders were more likely to receive advice on how to manage symptoms and receive medicine free of charge (92.7% vs. 83.8%) and less likely to attend the GP if asked to go to another healthcare service (1.5% vs. 3.7%) (Table 2). Responders were also more likely to have bought medication from the pharmacy (28.3% vs. 16.0%) and were less likely to have made an appointment with a GP (42.8% vs. 78.7%) if the service had not been available from the pharmacy (Table 3).

**Figure 1:**
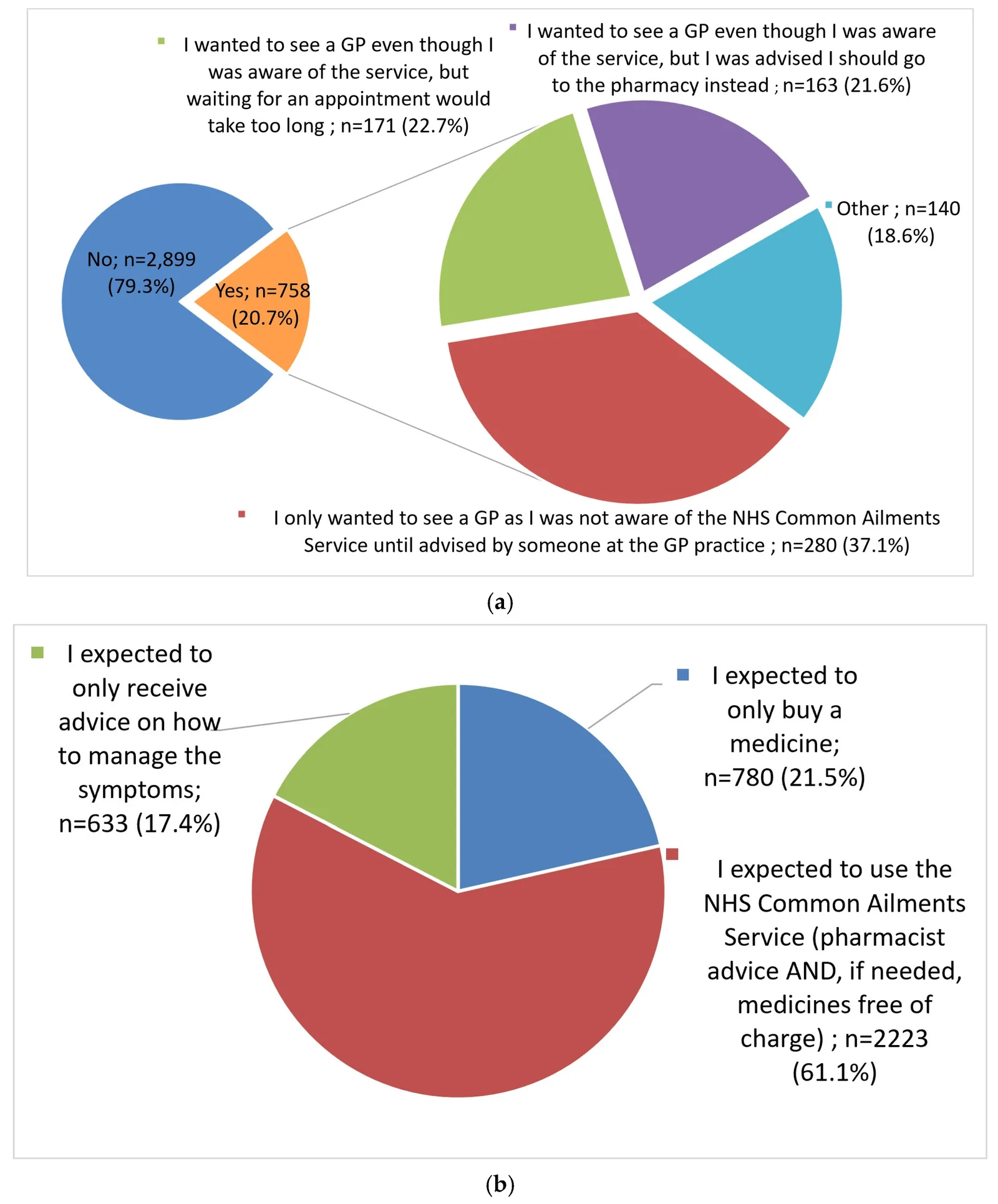
Responses to questions in relation to service engagement for consultations’ participants (or the person they care for) received under the Common Ailments Service, between 27th October 2025 and 31st March 2026 (*n* = 3660). (**a**) Did you—or the person you care for—try to see a GP before visiting the pharmacy? (missing data *n* = 2; 0.1%) If yes, what led to you—or the person you care for—being offered the NHS Common Ailments Service instead? (missing data *n* = 4; 0.5%) (**b**) What did you expect to happen when you—or the person you care for—visited the pharmacy today? (missing data *n* = 24; 0.7%).

Examining health-seeking behaviours amongst the cohort, 2899/3658 (79.3%) participants visited the pharmacy without trying to see a GP first (Figure 1a). When those who tried to see a GP first were asked why they visited the pharmacy, 280/754 (37.1%) stated they were not aware of the CAS until advised by someone at the GP surgery. Of the 334/754 (44.3%) who were aware of the service beforehand, 171/754 (22.7%) decided that waiting for a GP appointment would take too long and 163/754 (21.6%) were advised to go to the pharmacy by staff at the GP reception. Responses in the “other” category (*n* = 140/754) mainly referred to one of the three reasons as stated above (*n* = 114), with some additional explanation provided by patients in relation to supplementing a GP visit with a second opinion, further advice or treatment (*n* = 15), having had a good experience with the CAS and the pharmacy team previously (*n* = 10), and inclusivity (*n* = 1, no female doctor available at the surgery).

When visiting the pharmacy, 2223/3636 (60.7%) patients expected to use the CAS, 780/3636 (21.3%) only to buy medicine and 633/3636 (17.3%) only to receive advice on how to manage the symptoms (Figure 1b). From the 1411 responses of patients not expecting to access the CAS, 670 added a comment explaining why they received the service after all. The majority explained that they were not aware of what the service entailed and it was offered to them when discussing the symptoms with the pharmacy team (*n* = 550/670, 82.1%) or it was recommended so that the pharmacist would be able to access their medical record and check that current medication would not be interacting or contraindicated with any supplied treatment (*n* = 29/670, 4.3%). Fifteen patients (2.2%) were referred by another healthcare setting (general practice *n* = 13, optician *n* = 3, out-of-hours *n* = 1), and a further fifteen explained that the treatment they needed was not available to buy over the counter. Five patients (0.7%) explicitly mentioned that they could not afford the treatment they needed.

A total of 1567 (42.8%) of patients would have made an appointment with a GP had the service not been available from the pharmacy they visited, 1036 (28.3%) would have bought medication from the pharmacy, and 410 (11.2%) would have visited another pharmacy where the service would be available, whilst 146 (4.0%) would have done nothing and 159 (4.3%) would have looked on the internet for advice (Table 3). Emergency settings (Accident and Emergency, NHS 111, and out-of-hours) would have been patients’ first choice in 255 (7.0%) cases. Of the 2216 (60.5%) patients responding to the question about cost, 1769 (79.8%) stated that it was important/extremely important that any medicine they would receive would be free of charge, with 322 (8.8%) stating this was unimportant/extremely unimportant.

### 3.2. Service Outcomes and Patient Experience

Most patients (*n* = 3583, 97.8%) were managed in the pharmacy, with the majority (*n* = 3394, 92.7%) receiving advice and treatment as part of CAS (Table 2). Of the 65 (1.8%) who were referred to another healthcare professional, 54 (83.0%) were asked to see a GP, 5 (7.7%) an optometrist, and 3 (4.6%) to attend an accident and emergency department at a hospital or ring NHS 111. In the “other” category, one patient explained they were asked to see another pharmacist instead, who would be able to prescribe a treatment outside of CAS for them.

In terms of their experience during the CAS consultation, 3480 (95.1%) patients rated their overall experience with the service as excellent (Table 4). Patients agreed/strongly agreed to the following: the pharmacist explained the service to them in a way that they could understand (3594, 98.2%), they were involved as much as they wanted to be in decisions made about their care (3595, 98.2%), they had the opportunity to ask questions or raise concerns related to the service (3597, 98.3%), they were listened to (3620, 98.9%), and they were satisfied with the advice the pharmacist provided on how they can manage their symptoms after they leave the pharmacy (3599, 98.3%) and how the pharmacist explained what to do if their symptoms worsen (3563, 97.3%). Looking at health-seeking behaviour after speaking to the pharmacist, 2730 (74.6%) patients agreed/strongly agreed that they understand if they should manage similar symptoms in the future without having to see a pharmacist or a GP (by buying medicines or self-care) and 2905 (79.4%) that they are happy to do so. In cases where patients realised that they should not manage the symptoms themselves, 3114 (85.1%) agreed/strongly agreed that they would return to the pharmacy instead of trying to see a GP (Table 4).

Even though satisfaction was consistently very high, overall experience with the CAS consultation (excellent/good vs. fair vs. poor/very poor) was seen to vary significantly by the result of the consultation with the pharmacist (chi-squared test = 182.42, *p* < 0.001), where there was a higher rate of satisfaction (excellent/good) in patients who received advice and medicine (99.3%) compared to those who received advice only (95.8%) and those who referred to another healthcare service or professional (90.8%) (Supplementary Table S2). No associations were seen between satisfaction and importance of the medicine being free of charge, by age groups, or type of common ailment, where satisfaction was consistently between 98–99%.

### 3.3. Free-Text Responses

A total of 1499 free-text comments were left by participants in the data collection period. Content analysis in terms of experience returned 1151 occurrences of terms such as excellent (*n* = 333), great (*n* = 175), thank (*n* = 151), friendly (*n* = 125), happy (*n* = 100), brilliant (*n* = 78), fantastic (*n* = 72), amazing (*n* = 68), and efficient (*n* = 49). Overall, comments were extremely positive (*n* = 1484), as exemplified by one participant:

*“It’s [Common Ailments Service] worth its weight in gold—we’re so fortunate to have this service…”*

(Survey (S) 98, Dec 25)

**Figure 2:**
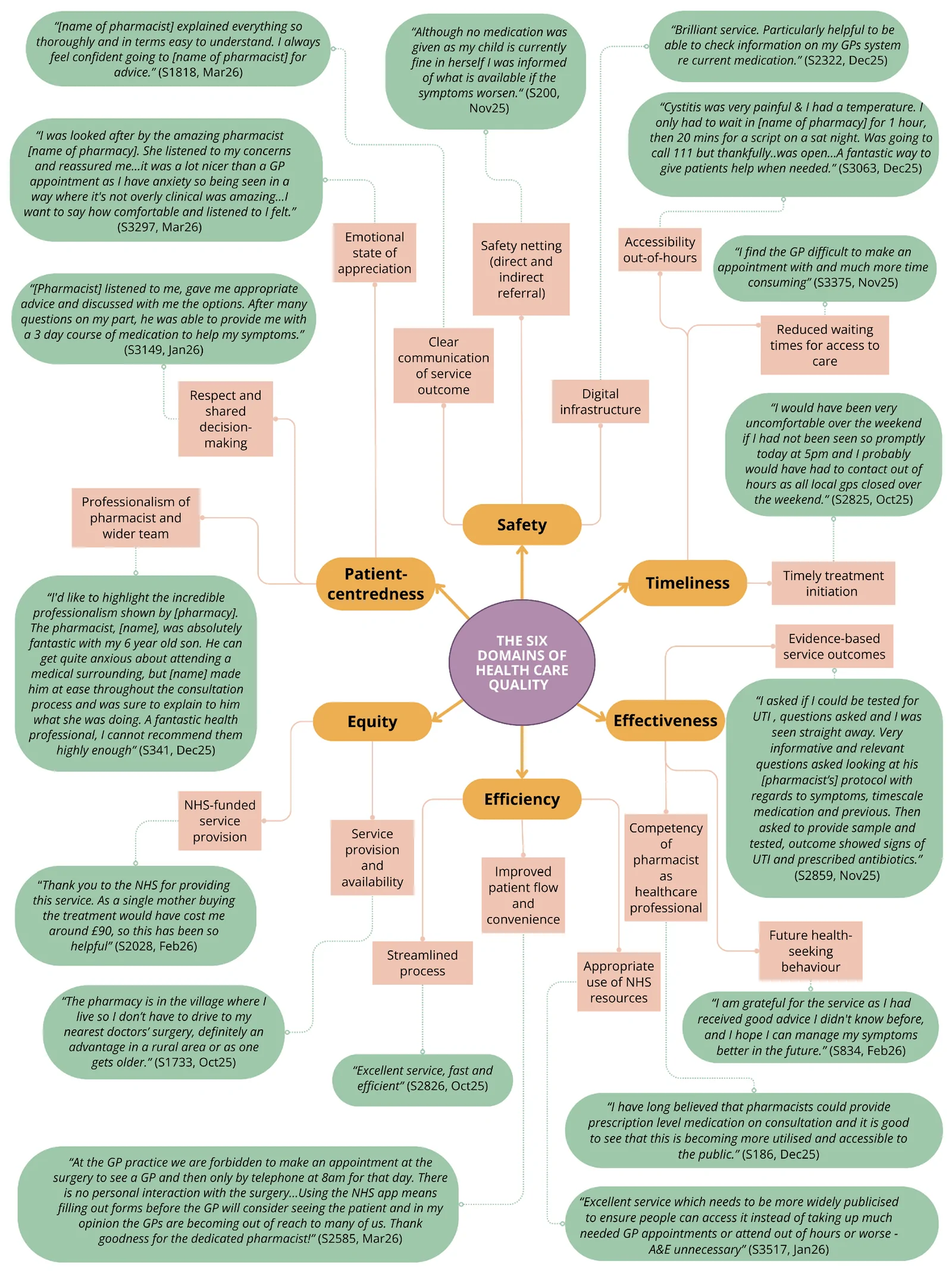
A schematic diagram displaying the framework analysis of free-text responses of inductively coded data from patient-reported experience measures (PREMs), collected from patients following common ailment (CAS) consultations between 27 October 2025 and 31 March 2026, mapped against the six domains of healthcare quality by the Institute of Medicine [17] (orange). Representative patient quotes from each subtheme (pink) within the domains are presented in green.

When a relationship was already established between the pharmacist and the patient before the consultation, indicated by several patients mentioning pharmacists by name and referencing past interactions, the sense of positive patient experience came across stronger. Inductively coded data were mapped against all six domains of healthcare quality, highlighting the holistic impact of the service implementation on patients (Figure 2). No additional themes were found using inductive analysis.

Safety: Patients detailed numerous examples of how any service outcome, including advice with and without treatment, had been communicated with them clearly by the pharmacists, ensuring they were confident and knew exactly how to manage their current symptoms, even when no medication was provided. They also discussed how safety netting was made explicit to them, with advice on worsening symptoms and when these would mean re-attendance at the pharmacy, a general practitioner, or an emergency care setting. Patients believed that the digital infrastructure (*Choose Pharmacy* platform) used to deliver the service reassured them their care would be safe, by allowing access to medical records when needed for decision-making. One patient noted that recording the consultation on the platform was a time-consuming process.

Effectiveness: Multiple comments on how pharmacists using sets of questions on the consultation room computer screen and using the links on the IT platform to show them details and information on treatments available indicated that the structured service pathways and digital infrastructure were perceived as an extremely effective way of providing standardised protocols for taking a targeted history, exploring symptoms, and providing evidenced-based service outcomes, whether that was advice only, or supplemented by a treatment choice. In a couple of cases only, patients expressed frustration that the treatment they wanted was not covered by the service’s formulary. Pharmacists were almost universally viewed as a competent healthcare professional that can deliver such a service in an effective way, with a single patient strongly disagreeing. The whole strong feeling of the service’s effectiveness fed into patients’ perceived future health-seeking behaviour, with multiple examples of patients confirming they now understand how to self-care and when to seek advice if they experience similar symptoms again, and if the latter, how they would choose to attend a pharmacy rather than general practice.

Patient centredness: Patients frequently commented on the professionalism of the pharmacist and the wider pharmacy team, discussing cases when they, or the person they cared for, had been provided with a personalised, compassionate, and holistic service, despite the business of the pharmacy environment. Many patients relayed a strong sense of an emotional state of appreciation, explaining how they felt listened to and cared for, not being judged, reassured, and that their fears or anxiety about seeing a healthcare professional were alleviated. They talked about the respect they received for their needs, highlighting how all efforts were made to ensure conversations were held in a confidential space. Their perspective was taken into account and the decision around service outcome options (choice of advice only vs. advice and treatment) was guided by their preferences. Only two examples were provided of when this was not followed: where a patient had not been informed that the discussion they were having with the pharmacist was under the Common Ailments Service and when the patient had not been informed outcomes of the service would be shared with their GP.

Timeliness: Patients relayed a very strong feeling of how the CAS ensured reduced waiting times for access to care compared to general practice, with patients explaining that even when they managed to get an appointment with a GP, this required a lengthy process. Numerous patients commented on how important it is to have the service available at times when other care settings, such as general practice or emergency care, are unavailable, for example outside business hours during weekdays or on weekends. The timely treatment initiation when needed was mentioned frequently, with examples of patients being in pain and recognising that any delays in treatment would have resulted in adverse outcomes for them, such as uncomplicated urinary tract infections leading to kidney infections.

Efficiency: It was widely perceived that the service’s delivery model of pharmacists being the first port of call with no prior appointment needed was extremely convenient and efficient in improving patient flow between care providers, with multiple experiences shared of frustrating attempts to get an appointment with a GP in the near future, or at all. The whole process was perceived as streamlined, with many patients commenting on how quick and efficient the journey was from deciding to seek help in a pharmacy to them being seen and receiving the care they needed. A handful of patients highlighted some challenges with the referral channels between GPs and pharmacies, with some examples provided of practice staff referring patients who did not meet the eligibility criteria to be consulted under CAS. Despite these isolated comments, there was a strong perception that the service was an appropriate use of NHS resources, focussing the management of minor ailments in pharmacies and enabling GPs to manage more complex patient cases. Many patients commented that the service should be expanded to include other conditions and that, for maximum efficiency, it should be publicised much more, as awareness for the service and the conditions it covers is limited among the public.

Equity: Patients often talked about how the service being funded under the NHS Wales ensured they were being provided with care, regardless of their financial situations. Examples were mentioned whereby the provision of treatment free of charge to the individuals ensured they could continue meeting their basic needs, such as obtaining food for themselves and their families. Consistent service availability irrespective of geographic location or personal skills was highly praised as well, with patients described how invaluable the service is in rural areas, especially when some surgeries have closed down, and how this is particularly helpful for people who do not drive. A very small number of patients mentioned challenges with identifying a pharmacy that would offer the service, as no official channels with live service provision exist.

## 4. Discussion

This study aimed to, for the first time, evaluate at large scale the patient-reported experience following a community pharmacy-based Common Ailments Service consultation with information collected directly from patients and to determine whether it is feasible to use a manual pharmacist-led recruitment model for continuous sustainable evaluation. While the data captured from 3660 surveys provided high-quality insight into the experience of patients using this Welsh community pharmacy service, the methods used to recruit patients and provide access to the survey were not deemed to be feasible owing to the overall low response rate. Nevertheless, high satisfaction levels were reported, with 95.1% overall patient experience ranked as excellent. The theory-based analysis against the six domains of healthcare quality [16] confirmed the quality of current provision of care, with comments mapped against all six domains, and identified some areas for improvement.

The technical feasibility of using QR codes as a scalable mechanism to capture and transmit patient-reported data digitally has been demonstrated, with more than 3600 responses received in the data collection period. However, the operational feasibility, involving routine integration into care workflows with sustainable pharmacist and patient engagement, remained challenging, with an overall response rate of 3.2%—lower than the 6.4% reported in the literature [13]. In its current form, the ‘proof of concept’ of the pharmacist-led recruitment and QR codes for digitally capturing PREMs piloted in this study is not a suitable model for sustainable evaluation.

The low response rate may have been due to a combination of low patient engagement and declining active pharmacist engagement within a high-pressure clinical environment, displaying the promotion leaflets in the consultation rooms and endorsing consultations with the code “PREMs” but not proactively signposting patients to the survey. Studies reporting high feasibility (completion rates exceeding 80%) shared several features not incorporated in the proof-of-concept approach in this study: seamless electronic health record integration with automatic data flow [14,19,20], dedicated implementation support including champions and training [15,21,22], and kiosks for in situ collection with staff assistance when needed [14,15,20,21]. A feature acting as an enabler of high response rates seen in the literature and adopted by our design was brief instruments requiring minimal time [12,14,23]. The common thread in studies reporting low feasibility (response rates below 30%) was not patient refusal of digital formats per se, but rather structural and procedural barriers that accumulated to prevent participation, with examples relevant to our approach comprising absence of electronic health record integration requiring manual processes [24,25,26,27], post-consultation or remote digital-only delivery without reminders [11,13,24], and limited organizational support in terms of implementation or unclear purpose of the long-term goal of the study [24,26,28]. In addition, the feasibility of collecting PREM digitally via a QR code varied by population characteristics, with age representing a clear gradient in the literature, similar to the findings of this study. Older adults have consistently reported lower digital confidence [29], were less likely to be aware of or helped with online services [29], and faced greater technical difficulties requiring assistance [14,30]. However, when provided with training and support, older patients in several studies successfully used digital tools and found them acceptable [21,31,32].

The high satisfaction levels reported in the study are in line with satisfaction frequently exceeding 80–90% in structured services and driven primarily by service-related attributes and staff characteristics, both within the UK [5,6,33,34,35] and at international level [36,37]. Patients highly valued the walk-in nature, extended hours, and convenient locations of community pharmacies, which present a significant advantage over scheduling appointments with general practitioners [5,33,35,38,39]. The professionalism of the whole team as well as the pharmacist’s friendliness, communication skills, perceived competence, and the ability to listen and provide clear explanations, building a trusting relationship, were highlighted [5,40,41,42,43,44,45].

A scheme constraint highlighted in the patient responses was the inappropriate referrals from practice staff, whereby eligibility criteria for the service had not been taken into account. Although the numbers in our findings are very small, the concept is in line with results from the England General Practice Community Pharmacist Consultation Service (incorporated into Pharmacy First since January 2024), where, even though 82.5% of patients were satisfied with the time the pharmacist spent with them and 80.9% with privacy, only 33.3% felt the consultation fully met their health needs, and 54.2% felt it was inappropriate that they had been advised to speak to a community pharmacist rather than a GP [46]. This suggests that the manner and context of referral from general practice to pharmacy may influence patient acceptance and satisfaction. Some other implementation considerations acted as barriers among participants to the wider use of the service, as reported elsewhere, such as a restricted formulary of available medications [47], service logistics that led to inconsistent availability [5], and a lack of public awareness about the service itself and the range of ailments it covers [5].

Understanding which healthcare services patients would have accessed in the absence of pharmacy schemes provides insight into the demand-management impact of these services. A total of 11.2% of patients in this study would have tried to find another pharmacy had CAS not been available. A further 42.8% and 7% of patients reported that they would have made an appointment with general practice or out-of-hours and emergency services, respectively, had CAS not been available, a very high rate consistent to those reported in the literature for services in Scotland [33], England [35,48], Canada [42,49], and Sweden [50]. A strong indicator of positive patient experience is the high likelihood of reusing the service, with patient-reported intention to reuse CAS for future minor ailments found to be 85.1%, similar to the high percentages reported in other studies, where measured [5,6,33,39,51]. These findings collectively indicate that pharmacy minor ailment schemes divert substantial demand from higher-cost healthcare settings and suggest that positive initial experiences with pharmacy-led care create a sustainable shift in future care-seeking behaviour away from general practice and emergency services toward community pharmacy as a first point of contact for self-limiting conditions and are in line with the overall strategic direction of the long-term NHS plan both in England and in Wales [52,53,54].

Only 28.3% of patients would have bought the medicine they needed had the service not been available, reinforcing the focus of the scheme on equity, particularly in supporting individuals who may struggle with the financial cost of purchasing treatment over the counter, further highlighted by free-text comments in the survey. Notably, the decommissioning of some minor ailment schemes in England in 2018 had raised serious concerns about protecting vulnerable populations [55]. However, 17.4% of patients in this study expected to only receive advice on how to manage the symptoms and 21.5% expected only to buy a medicine. More work is needed to better understand the balance between equity and the escalating costs of healthcare and inform policy changes.

The results of this study need to be contextualised within the limitations. The population sample was broadly representative of the total population having received a CAS consultation in terms of age and condition, but not in terms of service outcomes or actions had the service not been available. The survey was offered only online, with no incentives, and relied on patient self-selection. The response rate of 3.2% is lower than that reported elsewhere in the literature. Furthermore, using the number of consultations endorsed as PREMs to calculate the response rate is an assumption and not a certainty, as some patients may have been given survey information but the respective consultations were not endorsed as PREMs, and some pharmacists may have been endorsing some consultations as PREMs without actively sharing information about the survey with patients. Whilst the majority of pharmacies (97.9%) participated in the WIS, it was not possible to identify whether responses were received from people using every pharmacy, and this may limit the generalisability of the findings. The results may not be generalisable to health systems not like the UK’s where payer costs may be a factor in acceptability.

## 5. Conclusions

The findings of this study suggest that while PREMs collected through an online survey can capture high-quality insights, using clinicians to promote survey completion is unlikely to be feasible. A hybrid approach incorporating features such as integration in electronic health records with automated triggers and offering multiple completion modalities including in-pharmacy assistance may be more effective in capturing directly the reported patient experience of the service. However, the data captured indicate the Welsh community pharmacy CAS is highly valued, with 95.1% of patients reporting an “excellent” experience, and they suggest that a substantial number of the Welsh public view community pharmacy as the first port of call for common ailments. A theory-based analysis across the six domains of healthcare quality confirmed the high standard of care provision with the current service, primarily influenced by consultation quality, convenience, accessibility, and short waiting times; it also identified targeted areas for potential service enhancements such as the need for seamless referrals from other areas of primary care and consistent availability of the service across all pharmacies.

## Figures and Tables

**Table 1:** Number of consultations per month for patients in the sample in the current study (*n* = 3660) and the population cohort who had a Common Ailments Service consultation and endorsed with “PREMs” in the pharmacy between 27 October 2025 and 31 March 2026 (*n* = 114,216).

	Sample of Patients Completing the Survey (*n* = 3660) (%)	Population Cohort with CAS Consultation in the Pharmacy (*n* = 114,216) (%)	Response Rate
**Number of consultations**			
27/10/2025–31/10/2025	369 (10.1)	2213 (1.9)	16.7
01/11/2025–30/11/2025	441 (12.0)	15,350 (13.4)	2.9
01/12/2025–31/12/2025	1184 (32.3)	20,605 (18.0)	5.7
01/01/2026–30/01/2026	829 (22.7)	20,865 (18.3)	4.0
01/02/2026–27/02/2026	390 (10.7)	23,605 (20.7)	1.7
01/03/2026–31/03/2026	447 (12.2)	31,578 (27.6)	1.4

**Table 2:** Demographic characteristics, type of Common Ailments Scheme ailment, and key questions related to service outcomes of patients in the sample in the current study (*n* = 3660) and the total population cohort who had a Common Ailments Service consultation in the pharmacy between 27 October 2025 and 31 March 2026 (*n* = 240,058).

	Sample of Patients Completing the Survey (*n* = 3660) (%)	Total Population Cohort (*n* = 240,058) (%)
**Age of participant (or the person they care for) (*n* = 3626 responses)**		
0–9	311 (8.5)	38,318 (16.0)
10–15	207 (5.7)	19,914 (8.3)
16–17	72 (2.0)	6190 (2.6)
18–24	307 (8.4)	24,235 (10.1)
25–34	573 (15.7)	31,395 (13.1)
35–44	592 (16.2)	34,399 (14.3)
45–54	552 (15.1)	24,519 (10.2)
55–64	563 (15.4)	25,251 (10.5)
65–74	315 (8.6)	18,493 (7.7)
75–84	109 (3.0)	13,417 (5.6)
85+	25 (0.7)	3927 (1.6)
Missing	34 (0.9)	0 (0.0)
**Condition that CAS consultation was undertaken for** NB: For the question “What condition did you—or the person you care for—come to the pharmacy for”, additional clarification was sought from patients when the option “other” was selected. When the ailment described was clear and part of the Common Ailments Scheme, the respective count is included in the ailments presented below.		
Acne (acne vulgaris)	65 (1.8)	2767 (1.2)
Athlete’s foot	103 (2.8)	2666 (1.1)
Back pain	28 (0.8)	1753 (0.7)
Chickenpox	20 (0.5)	1586 (0.7)
Cold sores	10 (0.3)	706 (0.3)
Colic	0 (0.0)	12 (0.0)
Conjunctivitis	401 (11.0)	25,168 (10.5)
Constipation	92 (2.5)	7355 (3.1)
Diarrhoea	2 (0.1)	530 (0.2)
Dry eye	128 (3.5)	9434 (3.9)
Dry skin (including contact dermatitis and atopic eczema)	426 (11.6)	25,202 (10.5)
Haemorrhoids	46 (1.3)	2873 (1.2)
Hay fever (allergic rhinitis)	124 (3.4)	13,299 (5.5)
Head lice	71 (1.9)	11,029 (4.6)
Indigestion (dyspepsia)	194 (5.3)	7419 (3.1)
Ingrowing toenail	4 (0.1)	370 (0.2)
Intertrigo (red, inflamed skin in skin folds), tinea cruris and ringworm	173 (4.7)	10,279 (4.3)
Mouth ulcers	37 (1.0)	3123 (1.3)
Nappy rash	16 (0.4)	697 (0.3)
Oral thrush	100 (2.7)	5936 (2.5)
Scabies	111 (3.0)	18,633 (7.8)
Sore throat	594 (16.2)	32,551 (13.6)
Teething	8 (0.2)	383 (0.2)
Threadworm	132 (3.6)	25,475 (10.6)
Urinary tract infection	561 (15.3)	15,990 (6.7)
Vaginal (vulvovaginal) thrush	250 (6.8)	10,160 (4.2)
Warts and verrucae	72 (2.0)	4662 (1.9)
**Result of the consultation with the pharmacist (*n* = 3648 responses)** NB: If an option is not available through the CAS, this is marked as N/A		
Only received advice on how to manage the symptoms	189 (5.2)	25,055 (10.4)
Received advice on how to manage the symptoms AND received a medicine free of charge	3394 (92.7)	201,147 (83.8)
Asked to go to another healthcare service or professional	65 (1.8)	13,752 (5.7)
What healthcare service or professional were you—or the person you care for—asked to go to?		
GP	54 (1.5)	8765 (3.7)
Dentist	0 (0.0)	65 (0.03)
Optometrist	5 (0.1)	813 (0.3)
Accident and Emergency department at hospital	1 (0.0)	285 (0.1)
Out-of-hours (urgent medical care needed when regular GP surgery is closed)	0 (0.0)	N/A
NHS 111 (24/7 non-emergency healthcare service)	2 (0.1)	N/A
Other (another IP pharmacy, pharmacists, did not see a pharmacist)	3 (0.1)	3241 (23.6) NB: this includes out-of-hours and NHS 111
Refer patient (destination unknown)	N/A	583 (4.2)
Missing	12 (0.3)	0 (0.0)

**Table 3:** Key questions related to engagement with service of patients in the sample in the current study (*n* = 3660) and the total population cohort who had a Common Ailments Service consultation in the pharmacy between 27 October 2025 and 31 March 2026 (*n* = 240,058).

	Sample of Patients Completing the Survey (*n* = 3660) (%)	Total Population Cohort (*n* = 240,058) (%)
**What would the participant (or the person they care for) have done if the service had not been available from the pharmacy they visited** NB: If an option is not available through the CAS, this is marked as N/A		
Asked a friend for advice	14 (0.4)	N/A
Attended the Accident and Emergency department at hospital	33 (0.9)	135 (0.1)
Bought medication from the pharmacy	1036 (28.3)	38,350 (16.0)
Called NHS 111 for advice	92 (2.5)	1796 (0.7)
Done nothing	146 (4.0)	6141 (2.6)
Looked on the internet for advice	159 (4.3)	N/A
Made an appointment to use the out-of-hours service	130 (3.6)	2304 (1.0)
Made an appointment with a GP	1567 (42.8)	188,807 (78.7)
Made an appointment with a nurse of health visitor	28 (0.8)	439 (0.2)
Made an appointment with another healthcare professional (e.g., dentist or optometrist)	33 (0.9)	2057 (0.9)
Visited a pharmacy where the service was available	410 (11.2)	N/A
Missing	12 (0.3)	29 (0.0)

**Table 4:** Key questions related to patient-reported experiences for patients (or the person they care for) who had a Common Ailments Service consultation in the pharmacy between 27 October 2025 and 31 March 2026 (*n* = 3660).

	Excellent *n* (%)	Good *n* (%)	Fair *n* (%)	Poor *n* (%)	Very Poor *n* (%)	Missing/NA *n* (%)
**Overall experience with the NHS Wales Common Ailments Service consultation**	3480 (95.1)	26 (0.7)	134 (3.7)	5 (0.1)	7 (0.2)	8 (0.2)
	Strongly agree *n* (%)	Agree *n* (%)	Not sure *n* (%)	Disagree *n* (%)	Strongly disagree *n* (%)	Missing/NA *n* (%)
**Thinking back to the consultation with the pharmacist, how strongly do you agree with the statements below?** **If you are filling this is for someone else, please answer the questions from their perspective.**						
The pharmacist explained the service to me in a way that I could understand	3214 (87.8)	380 (10.4)	21 (0.6)	10 (0.3)	22 (0.6)	13 (0.4)
I was involved as much as I wanted to be in decisions made about my care	3227 (88.2)	368 (10.1)	34 (0.9)	4 (0.1)	10 (0.3)	17 (0.5)
I had the opportunity to ask questions or raise concerns related to the service	3187 (87.1)	410 (11.2)	27 (0.7)	9 (0.2)	11 (0.3)	16 (0.4)
I felt that I was listened to	3320 (90.7)	300 (8.2)	6 (0.2)	4 (0.1)	14 (0.4)	16 (0.4)
I am satisfied with the advice the pharmacist provided on how I can manage my symptoms after I leave the pharmacy	3337 (91.2)	262 (7.2)	16 (0.4)	12 (0.3)	14 (0.4)	19 (0.5)
I am satisfied with how the pharmacist explained what to do if my symptoms worsen	3235 (88.4)	328 (9)	47 (1.3)	13 (0.4)	19 (0.5)	18 (0.5)
**After speaking to the pharmacist and thinking about the next time you—or the person you care for—may have similar symptoms, how strongly do you agree with the statements below?** **If you are filling this is for someone else, please answer the questions from their perspective.**						
I understand if I SHOULD manage the symptoms without having to see a pharmacist or a GP (by buying medicines or self-care)	1943 (53.1)	787 (21.5)	513 (14.0)	216 (5.9)	124 (3.4)	77 (2.1)
If I SHOULD manage the symptoms without having to see a pharmacist or a GP, I am HAPPY to do so	2074 (56.6)	831 (22.8)	353 (9.6)	238 (6.5)	97 (2.7)	67 (1.8)
If I SHOULD NOT manage the symptoms myself, I will return to the pharmacy instead of trying to see a GP	2350 (64.2)	764 (20.9)	283 (7.7)	157 (4.3)	46 (1.3)	60 (1.6)

## Data Availability

The data presented in this study are available on request from the corresponding author due to privacy.

## Supplementary Materials

The following supporting information can be downloaded at https://www.mdpi.com/article/10.3390/pharmacy14050112/s1: Table S1: Surveys excluded from final dataset for analysis, stratified by frequency of ailment/condition/reason for visiting the pharmacy described in text provided in option “other” for the question “What condition did you—or the person you care for—come to the pharmacy for?” (*n* = 182); Table S2: Association of overall satisfaction with the NHS Wales Common Ailments Service consultation with importance of the medicine being free of charge, age groups, or type of common ailment.
