# Supplementary material for: “It’s Worth Its Weight in Gold”: Patient Reported Experience Measures of a National NHS-Funded Community Pharmacy-Based Common Ailments Service
Source: Pharmacy (Basel). 2026 Aug 3;14(5):112. doi: 10.3390/pharmacy14050112 (PMC13516897; doi:10.3390/pharmacy14050112)
Supplement: Supplementary file 1 [file pharmacy-14-00112-s001.zip › pharmacy-4417761-supplementary.pdf]

Supplementary Table S1: Surveys excluded from final dataset for analysis, stratified by frequency of ailment/condition/reason for visiting the pharmacy described in text provided in option "other" for the question "What condition did you - or the person you care for - come to the pharmacy for?" (n=182).
NB: Total percentages may not add up to 100 due to rounding

Condition	Count (%)
Ears	41 (22.5)
Infection - skin	34 (18.7)
Infection - eye	32 (17.6)
Clicked on Welsh RW code by mistake	11 (6.0)
Emergency Contraception	11 (6.0)
Flu vaccine	11 (6.0)
N=3 for each of:<br>No information provided/unsure of condition; Bug bite; Medication advice	9 (4.9)
N=2 for each of:<br>Crohn's/IBS; Infection – tooth; Hormonal contraception; Migraine; Reaction to prescribed medication; Shoulder impingement/pain; Stomach cramps; Weight management	16 (8.8)
N=1 for each of:<br>Arthritis flare up; Asthma; Chilblains; Chronic nasal congestion; Diabetes; Dizziness; Dysplasia; Emergency supply; End of life COPD; Epilepsy; Foot ulcer; Giddiness; Hard skin/ callous; Swelling on ankle; Treatment after tonsillectomy; Unknown pain in side; Varicose veins	17 (9.3)

Supplementary Table S2: Association of overall satisfaction with the NHS Wales Common Ailments Service consultation with importance of the medicine being free of charge, age groups, or type of common ailment.

	**Overall experience with the NHS Wales Common Ailments Service consultation**			
	**Excellent/Good n=3614 n (%)**	**Fair n=26 n (%)**	**Poor/Very poor n=12 n (%)**	**Chi-squared, p-value**
**What was the result of the consultation with the pharmacist?**				
Asked to go to another healthcare service or professional	59 (90.8)	0 (0.0)	6 (9.2)	**182.42, <0.001**
Only received advice on how to manage the symptoms	181 (95.8)	6 (3.2)	2 (1.1)	
Received advice on how to manage the symptoms AND received a medicine free of charge	3,368 (99.3)	20 (0.6)	4 (0.1)	
Missing	6	0	0	
**How important was it to you – or the person you care for – that you could receive a medicine free of charge**				
Important/extremely important	1,747 (98.8)	13 (0.7)	8 (0.5)	2.58, 0.63
Not sure	122 (97.6)	2 (0.6)	1 (0.8)	
Unimportant/extremely unimportant	317 (98.4)	2 (1.6)	3 (0.9)	
**Age groups (years)**				
0-15	510 (98.5)	7 (1.4)	1 (0.2)	18.38, 0.11
16-24	375 (98.9)	3 (0.8)	1 (0.3)	
25-34	567 (99.0)	5 (0.9)	1 (0.2)	
35-44	583 (98.6)	6 (1.0)	2 (0.3)	
45-54	547 (99.3)	2 (0.4)	2 (0.4)	
55-64	560 (99.6)	2 (0.4)	0 (0.0)	
65+	443 (98.7)	1 (0.2)	5 (1.1)	
**Ailment**				
Acne (acne vulgaris)	64 (98.5)	1 (1.5)	0 (0)	38.76, 0.77[a]
Athlete's Foot	27 (96.4)	0 (0)	1 (3.6)	
Back pain	20 (100.0)	0 (0)	0 (0)	
Chickenpox	10 (100.0)	0 (0)	0 (0)	

Cold sores	396 (99.0)	4 (1)	0 (0)
Colic	91 (98.9)	0 (0)	1 (1.1)
Conjunctivitis	1 (50.0)	1 (50)	0 (0)
Constipation	127 (99.2)	1 (0.8)	0 (0)
Diarrhoea	422 (99.3)	3 (0.7)	0 (0)
Dry eye	46 (100.0)	0 (0)	0 (0)
Dry skin	123 (99.2)	0 (0)	1 (0.8)
Haemorrhoids	69 (98.6)	0 (0)	1 (1.4)
Hay fever	1 (100.0)	0 (0)	0 (0)
Head lice	84 (97.7)	1 (1.2)	1 (1.2)
Indigestion (dyspepsia)	4 (100.0)	0 (0)	0 (0)
Ingrowing toenail	65 (100.0)	0 (0)	0 (0)
Intertrigo, tinea cruris and ringworm	37 (100.0)	0 (0)	0 (0)
Mouth Ulcers	16 (100.0)	0 (0)	0 (0)
Nappy Rash	100 (100.0)	0 (0)	0 (0)
Oral Thrush	107 (99.1)	1 (0.9)	0 (0)
Scabies	110 (99.1)	0 (0)	1 (0.9)
Sore Throat	586 (98.7)	5 (0.8)	3 (0.5)
Teething	8 (100.0)	0 (0)	0 (0)
Threadworm	129 (97.7)	3 (2.3)	0 (0)
Urinary tract infection	554 (98.8)	4 (0.7)	3 (0.5)
Vaginal (vulvovaginal) thrush	250 (100.0)	0 (0)	0 (0)
Warts and verrucae	71 (98.6)	1 (1.4)	0 (0)

a Chi-squared test based on ailments with 10 or more consultations
